# Higher amyloid deposition and lower white matter volume in cognitively healthy seniors exceeding French alcohol recommendations

**DOI:** 10.1016/j.ebiom.2026.106376

**Published:** 2026-07-15

**Authors:** Célia Soussi, Julie Gonneaud, Nicolas Cabé, Alice Laniepce, Natalie L. Marchant, Fabienne Collette, Robin de Flores, Emilie Foyard, Brigitte Landeau, Florence Mezenge, Gaël Chételat, Shailendra Segobin, Anne-Lise Pitel, Medit-Ageing Research Group, Eider Arenaza-Urquijo, Emiliano Albanese, Florence Allais, Claire André, Sebastian Baez-Lugo, Mohamed Bahi, Nikita Belly, Maelle Botton, Gaël Chételat, Anne Chocat, Fabienne Collette, Robin de Flores, Vincent de La Sayete, Marion Delarue, Stéphanie Egret, Rawda El Sadawy, Eglantine Ferrand Devoue, Eric Frison, Idir Hamdidouche, Marc Heidmann, Thibaut Jorand, Agathe Joret, Perla Kaliman, Olga Klimecki, Elizabeth Kuhn, Brigitte Landeau, Julie Lebahar, Gwendoline Ledu, Valérie Lefranc, Antoine Lutz, Marine Manard, Natalie L. Marchant, Sara Martinez, Florence Mezenge, Laurence Michel, Inès Moulinet, Valentin Ourry, Christophe Philips, Géraldine Poisnel, Anne Quillard, Géraldine Rauchs, Stéphane Rehel, Charlotte Reid, Laura Richert, Eric Salmon, Corinne Schimmer, Siya Sherif, Delphine Smagghe, Rhonda Smith, Clémence Tomadesso, Edelweiss Touron, Patrik Vuilleumier, Cédric Wallet, Caitlin Ware, Miranka Wirth

**Affiliations:** a Normandie Univ, UNICAEN, INSERM, UA20, NeuroPresage, Cyceron, 14000, Caen, France; b Service d’Addictologie, Centre Hospitalier Universitaire de Caen, France; c Normandie Univ, UNIROUEN, CRFDP (EA 7475), Rouen, France; d Division of Psychiatry, University College London, London, United Kingdom; e GIGA-CRC, In Vivo Imaging, Université de Liège, Liège, Belgium; f Belgian National Fund for Scientific Research (F.R.S.-FNRS), Brussels, Belgium; g Normandie Univ, UNICAEN, PSL Université, EPHE, INSERM, U1077, CHU de Caen, GIP Cyceron, NIMH, 14000, Caen, France; h Institut Universitaire de France (IUF), 75005, Paris, France

**Keywords:** Alcohol consumption, Older adults, Structural MRI, 18F-florbetapir PET, Alzheimer's disease, Public health guidelines

## Abstract

**Background:**

Older adults are particularly vulnerable to alcohol's effects. Our aim was to establish whether differences in neurocognitive health exist between cognitively healthy seniors, depending on whether they follow recent French health recommendations for stricter drinking limits beyond age 65.

**Methods:**

We conducted a retrospective cross-sectional study including 133 community-dwelling cognitively healthy participants aged over 65 from the Age-Well Study. Based on their compliance with recommendations, participants were classified as lower-risk or higher-risk. Neurocognitive health was assessed comprehensively through standard cognitive testing, and voxel-wise analyses of structural integrity and cortical amyloid PET burden.

**Findings:**

Higher-risk participants displayed more neocortical amyloid deposition (*F* = 7.577; *p* = 0.007; *d =* 0.553) and lower cerebral volume, in the absence of a difference in cognition (*p* > 0.1).

**Interpretation:**

These results highlight that alcohol consumption exceeding age-specific recommendations is associated with lower brain integrity, notably including elevated amyloid burden, a biomarker strongly linked with higher risk of Alzheimer's disease–related cognitive decline and dementia. Our findings emphasise the importance of strict adherence to lower-risk thresholds in older populations.

**Funding:**

European Union’s Horizon 2020 Research and Innovation Programme (No 667696); 10.13039/501100001677Institut National de la Santé et de la Recherche Médicale (INSERM); Fondation Entrepreneurs MMA; 10.13039/501100014439FONDATION ALZHEIMER; 10.13039/501100018696Région Normandie; 10.13039/501100021790Fondation Recherche Alzheimer; 10.13039/501100003750Association France Alzheimer; 10.13039/501100004431Fondation de France; 10.13039/501100020320Institut pour la Recherche en Santé Publique (IRESP).

**Research in context**

**Evidence before this study**

We searched PubMed for articles written in English and published on or before December 2025. The search terms used were combinations of the following: ‘alcohol’, ‘drinking’, ‘ethanol’, ‘older adults', ‘ageing’, ‘cognition’, ‘dementia’, ‘neurocognitive disorder’, ‘neuroimaging’, ‘amyloid’, and ‘Alzheimer's disease’. Extensive evidence exists to demonstrate the adverse effects of heavy alcohol consumption on neurocognitive health. Nevertheless, the findings related to low-to-moderate consumption are inconsistent due to a number of methodological limitations. Neuroimaging studies assessing the relationship between alcohol consumption and amyloid burden remain limited. No study has operationalised alcohol consumption according to adherence to age-specific recommendations.

**Added value of this study**

This study is one of the few to examine neurocognitive health, structural brain integrity, and cortical amyloid burden in cognitively healthy older adults, according to adherence to age-specific national alcohol consumption guidelines. This study utilised a well-characterised community-based cohort and multimodal neuroimaging techniques to reveal that individuals who exceed recent French recommendations for adults aged over 65 years exhibit altered cerebral integrity despite preserved cognitive performance. It is noteworthy that the present study provides evidence of greater neocortical amyloid deposition associated with higher-risk alcohol consumption.

**Implications of all the available evidence**

The findings of this study indicate that exceeding age-specific recommendations for alcohol consumption is associated with poorer brain integrity, including neuropathology related to Alzheimer's disease, even in older adults without cognitive impairment. The findings of this study lend support to the implementation and dissemination of alcohol consumption guidelines that are age-specific. The results may encourage stricter adherence to alcohol guidelines among older adults, but also call for more comprehensive communication with the general population and healthcare workers about the increased risk associated with alcohol consumption as people age.

## Introduction

Vulnerability to alcohol increases with age.^1^ A reduction in enzymatic activity is observed in the gastric and hepatic systems,^2^^,^^3^ altering alcohol metabolisation. Ageing is also associated with a reduced ratio of lean body mass to fat mass and with a lower proportion of body water, reducing the volume of alcohol distribution. These physiological changes result in higher blood concentration of ethanol for an equivalent dose.^4^ Overall, older adults are exposed to increased risks associated with alcohol consumption, notably regarding falls and related injuries, cardiovascular health,^5^ liver health,^6^ sleep disorders,^7^ drug interactions,^8^ and cognitive impairment.^9^^,^^10^

There is vast evidence of the detrimental effects of excessive alcohol consumption on cerebral integrity, cognition, risk of overall dementia and Alzheimer's disease in the general population of middle-aged and older adults.^9^, ^10^, ^11^, ^12^, ^13^ However, there is surprisingly no consensus regarding low-to-moderate alcohol consumption on neurocognitive health, with data indicating either a linear relationship (i.e., a dose–response harmful effect)^14^, ^15^, ^16^, ^17^, ^18^ or a J-shaped relationship (i.e., a “protective” effect of low-to-moderate alcohol consumption).^9^^,^^11^, ^12^, ^13^^,^^19^, ^20^, ^21^, ^22^, ^23^, ^24^ These apparent protective effects may actually result from several methodological biases: survival bias, inclusion of former drinkers in the abstinent group and confounding variables.^21^ The latter is even more important since studies show that older adults with low-to-moderate consumption often have higher levels of education^14^^,^^25^, ^26^, ^27^ and socio-economic levels^26^^,^^28^—two proxies of cognitive reserve— than abstainers. Cognitive reserve may therefore play a key role in the relationship between alcohol consumption and neurocognitive measures.^14^^,^^25^ Other potentially confounding variables are of importance, such as psychiatric symptoms,^29^, ^30^, ^31^, ^32^, ^33^ use of medical treatment,^8^ smoking^34^^(preprint)^ or *APOE* ε4 status.^35^ The latter should notably be controlled in studies investigating links between alcohol and amyloid burden, given the strong association between this genetic risk factor of Alzheimer's disease and amyloid accumulation.^36^

Age-specific health guidelines regarding alcohol consumption have only been proposed in the US^37^^,^^38^ and, more recently, in France.^39^ New French guidelines regarding alcohol consumption for adults aged 65 and over define a low-risk alcohol consumption below 2 standard drinks (one standard drink = ∼10g of pure ethanol in France) per occasion and 7 standard drinks per week (compared to 10 standard drinks per week for adults under the age of 65, or for all groups before 2022).^39^ As it is now admitted that there is no safe level of alcohol consumption for global health,^40^ these thresholds are meant to define a lower risk. These recommendations are an interesting tool to define groups of drinkers as they allow higher-risk consumption to be defined by both weekly and per occasion intake, as opposed to previous studies, which often focus solely on the number of drinks per week. In a context of a persistent lack of consensus regarding the impact of low-to-moderate alcohol consumption, this study aims to establish whether differences in cognition, cerebral integrity and amyloid burden exist in cognitively healthy older adults, depending on whether they follow or not French public health recommendations regarding low-risk alcohol consumption thresholds in seniors. To control for potentially confounding variables, we investigated whether groups differed in terms of demographics, anxiety and depressive symptoms, genetic risk for Alzheimer's disease (*APOE*4 status), lifestyle factors that could influence cognitive reserve, smoking or medical treatments.

Given the lack of consensus in the literature, we cautiously hypothesised that exceeding recommended thresholds would be associated with some subtle but detectable differences in neurocognitive integrity, even within a cognitively healthy population, reflected by lower cognitive performance and/or lower brain integrity in participants with higher-risk alcohol consumption.

## Methods

### Participants

Of 157 participants initially assessed, 137 community-dwelling cognitively healthy older adults were enrolled in the Age-Well randomised controlled trial of the Medit-Ageing European project (NCT02977819).^41^ Baseline data of 133 participants were included in the present study (Supplementary Figure S1). The details of the inclusion and exclusion criteria are described in a previous publication^41^ and are listed in the Supplementary Materials. Participants underwent an assessment that included a comprehensive behavioural and neuroimaging evaluation within a maximum period of 2 months from November 2016 until April 2018.

### Measurements

For all participants, biological sex and education level (in years) were self-reported at inclusion.

#### Alcohol consumption

Participants completed a self-administered questionnaire assessing their alcohol consumption over the past 12 months. They reported the frequency of their alcohol consumption and the typical quantity of standard drinks of alcohol consumed per drinking occasion over the past year. Based on these answers, the individual number of drinks per week was computed by multiplying the frequency by the typical quantity consumed per occasion.

Participants were divided into two groups (lower-risk and higher-risk) based on current French governmental recommendations (2022) on alcohol drinking for adults aged 65 and over.^39^ These recommendations have been elaborated using opinions and recommendations from the *Société française de gériatrie et de gérontologie* and the *Société française d'alcoologie*,^42^ *Santé publique France*,^39^ the *American Geriatric Society*^37^ and the *National Institute on Alcohol Abuse and Alcoholism* in the U.S.^38^ The recommendations include two criteria: one related to the number of drinks per occasion (≤2 drinks per occasion) and another one related to the number of drinks per week (≤7 drinks per week).

#### Cognition

Neuropsychological evaluation encompassed standardised assessments of episodic memory, executive functioning and attention-processing speed. For each domain, a composite score was computed by averaging outcome measures of each neuropsychological test using the mean and standard deviation of the entire group (Z-scores).

A verbal episodic memory composite score was created from two established measures of episodic memory: The California Verbal Learning Test, second edition (CVLT-II) and the Wechsler Memory Scale (WMS) IV Logical Memory, Story B. We first standardised the scores for each of their constituent parts. For the CVLT-II, this included the sum of trials 1–5, immediate free recall, and delayed free recall. For Logical Memory, this included immediate and delayed free recalls. The unweighted average of these five z-scores yielded the verbal episodic memory composite score.

An executive functioning score was computed by averaging the standardised scores of Wechsler Adult Intelligence Scale IV (WAIS IV) Digit Span backward (raw score),^43^ Trail Making Test Part B (response time), Stroop interference index (response time for interfering-neutral items) and Letter Fluency (raw score).^44^

An attention-processing speed composite was computed by averaging the standardised scores on the Trail Making Test Part A (response time), Stroop naming (response time), WAIS IV Digit Span forward (raw score) and Coding (raw score).^43^

Trail Making Test and Stroop scores were reversed so that higher individual scores and therefore higher total compound scores indicate better performance.

#### Cerebral integrity

##### Acquisition

All participants were scanned using the same magnetic resonance imaging (MRI; Philips Achieva 3.0T scanner) and positron emission imaging (PET; Discovery RX VCT 64 PET-CT scanner, General Electric Healthcare) cameras at the Cyceron Center (Caen, France).

A high-resolution T1-weighted structural image was acquired using a 3D fast-field echo sequence (sagittal; repetition time (RT), 7.1 ms; echo time (ET), 3.3 ms; flip angle, 6°; field of view (FOV), 256 × 256 mm^2^, 180 slices, voxel size: 1 × 1 × 1 mm^3^) and a 3D fluid-attenuated inversion recovery (FLAIR; sagittal; RT, 4800 ms; ET, 272 ms; inversion time, 1650 ms; flip angle, 40°; FOV, 250 × 250 mm^2^, 180 slices, voxel size: 0.98 × 0.98 × 1 mm^3^).

Then, an echo-planar imaging/spin echo diffusion weighted sequence (DKI) was performed at multiple shells: 3b-values (0, 1000, 2000 s/mm2) (axial; 30 directions; TR = 6100 ms; TE = 101 ms; FOV, 216 × 216 mm^2^, 48 slices, voxel size: 2.7 × 2.7 × 2.7 mm^3^) and additional blips images with b = 0 s/mm 2 (number of signal averages, 9) were acquired in the reverse phase encoding direction for susceptibility distortion.

18F PET (Fluorine-18 florbetapir positron emission tomography) scans were acquired with a resolution of 3.76 × 3.76 × 4.9 mm^3^ (field of view = 157 mm). Forty-seven planes were obtained with a voxel size of 1.95 × 1.95 × 3.27 mm^3^. A transmission scan was performed for attenuation correction before the PET acquisition. A 10-min scan was acquired 50 min after the intravenous injection of ∼4MBq/kg of Florbetapir.

##### Preprocessing

Using SPM12, T1-weighted images, aided by corresponding FLAIR images, were segmented into grey matter, white matter and cerebrospinal fluid and normalised to the Montreal Neurological Institute (MNI) space. Grey matter normalised segments were modulated to preserve brain volumes and the resultant images were smoothed using an 8-mm full-width half-maximum (FWHM) Gaussian kernel. Unmodulated images were averaged and thresholded at 0.5 to create grey matter and white matter binary masks for statistical analyses.

DKI images underwent correction for susceptibility artefacts, eddy current distortions, and subject motion using Functional MRI of the Brain diffusion toolbox (FSL 5.0.9). DKI data were processed using MATLAB R2012b (MathWorks) and diffusional kurtosis estimator software (DKE, version 2.6) to estimate the diffusional kurtosis tensor. Images were smoothed using a 3.375 × 3.375 × 3.375 mm FWHM Gaussian filter. Mean diffusion (MD) and fractional anisotropy (FA) maps were extracted from the DKE, co-registered to the T1-weighted MRI, and normalised to the MNI template by applying the deformation parameters from the corresponding T1-weighted MRI. The resultant images were further smoothed with a 6.7-mm FWHM Gaussian kernel, so that the final smoothness of DKI images was the same as anatomical MRI images (8 × 8 × 8 mm^3^). Images were then masked to exclude non-white matter voxels from the analyses.

18F-PET images were co-registered onto their corresponding MRI and normalised to the MNI template using deformation parameters from the T1-weighted segmentation procedure. Images were then quantitatively normalised using the cerebellar grey matter as the reference region, resulting in standardised uptake value ratios (SUVRs). Images were smoothed using a 10-mm FWHM Gaussian kernel for voxel-wise analyses. A neocortical mask including the entire grey matter, except the cerebellum, occipital and sensory motor cortices, hippocampi, amygdala and basal nuclei^45^ was applied during statistical analyses. Neocortical SUVr values were also transformed to Centiloids^46^ (see Supplementary Method for details). PET data and DKI images were missing for one participant in the lower-risk group.

#### Potentially confounding variables

*APOE* genotype was determined using a standardised protocol involving a polymerase chain reaction-based assay that uses HhaI restriction enzymes to digest the PCR products, and separates the resulting digestion fragments by electrophoresis on polyacrylamide gels.^47^ To establish ***APOE4* status**, *APOE4* carriers were defined as carriers of one or two ε4 alleles. Individuals with no ε4 allele were defined as *APOE4* non-carriers.

Regarding psychiatric symptoms, depression symptoms were measured with the Geriatric Depression Scale (GDS).^48^ Trait anxiety was measured with the State-Trait Anxiety Inventory B (STAI-B).^49^

To assess lifestyle factors that could influence cognitive reserve, the Lifetime of Experience Questionnaire (LEQ)^50^ was used. The LEQ was adapted, harmonised, and validated across France, Germany, Spain and the United Kingdom in the context of the EU-funded project Medit-Ageing (Age-Well and SCD-Well). This questionnaire was used to assess participants’ engagement in complex mental activities through three life periods: young adulthood (13–30 years), midlife (30–65 years), and late-life (since 65 years or since retirement). The LEQ total score was obtained by adding the scores across all life periods; a higher score represents higher engagement in complex mental activities.

Smoking habits were assessed in accordance with the World Health Organisation's guidelines on tobacco questions for monitoring surveys. People reported whether they currently, previously, or never smoked. This allowed identification of the proportion of current smokers and former smokers amongst participants.

The number of medical treatments used by participants was quantified by category: cardiovascular (including antihypertensive, anticoagulant/antiplatelet, diabetes treatment, hypoglycaemic treatment and cholesterol-lowering treatment), anti-inflammatory treatment and hormone replacement therapy for thyroid and for menopause.

### Ethics

All participants provided written informed consent for the study, and the trial was approved by the ethics committee (Comité de Protection des Personnes Nord-Ouest III, Caen, France; trial registration number: EudraCT: 2016-002441-36; IDRCB: 2016-A01767-44). This study was carried out in line with the Declaration of Helsinki (2024).

### Statistics

Between-group differences in demographics and other potential confounding variables were described using chi2, Student's t-tests or Wilcoxon rank sum.

We evaluated between-group differences on cognitive measures using Type III ANCOVAs, adjusting for sex, age, and level of education. Exploratory analyses were also carried out on all the cognitive scores used to compute the composite scores. For Centiloid values, the same model was extended by adding *APOE*4 status as an additional covariate. For each model, the assumptions of ANCOVAs were met, except for Centiloid values (Shapiro–Wilk test: *W* = 0.853, *p* < 0.001; Levene's test: *F* = 5.427, *p* = 0.021). For the latter, we additionally performed a permutation-based ANOVA (5000 permutations) using the Freedman–Lane method to account for covariates under non-normality conditions.

Statistical analyses were carried out using R Statistical Software (v4.2.3). Significant results are reported at a 5% alpha-level (*p* < 0.05).

We investigated between-group differences in grey matter and white matter volume in a voxel-by-voxel approach, using SPM12 *two-sample t-tests* with age, sex, and level of education as covariates. A similar model was applied to 18F-PET images with *APOE*4 status as an additional covariate. We applied a higher-risk < lower-risk contrast for all structural MRI images, testing for lower volume in the higher-risk group, and a lower-risk < higher-risk contrast for 18F-PET images, testing for more amyloid deposition in the higher-risk group. We also examined the reverse contrasts to confirm the absence of significant findings in the opposite direction (data not shown). Analyses were performed using the TFCE (Threshold-Free Cluster Enhancement) method implemented in the SPM toolbox (https://github.com/ChristianGaser/tfce), with 5000 permutations for non-parametric significance testing. Results are reported at an uncorrected *p* < 0.005 threshold (*p* < 0.005 TFCE), and corrected for multiple comparisons using the family-wise error (FWE) rate at *p* < 0.05 (*p* < 0.05 TFCE, FWE-corrected).

When between-group differences were observed, we conducted regression analyses in the higher-risk group to determine whether a linear dose–response relationship existed between the number of drinks consumed per week and the concerned measures: cognitive composite scores, Centiloid, total volume from all significant clusters identified in the higher-risk < lower-risk contrast or mean standardised uptake value ratios (SUVRs) from all significant clusters identified in the lower-risk < higher-risk contrast. Analyses were corrected for age, sex, education, as well as total intracranial volume for grey and white matter and *APOE*4 status for Centiloid and SUVRs.

### Role of funders

The Age-Well randomised clinical trial is part of the Medit-Ageing project and is funded through the European Union's Horizon 2020 Research and Innovation Program (grant 667696), Institut National de la Santé et de la Recherche Médicale, Région Normandie, and Fondation MMA des Entrepreneurs du Futur.

The funders and sponsors had no role in the design and conduct of the study; collection, management, analysis, and interpretation of the data; preparation, review, or approval of the manuscript; and decision to submit the manuscript for publication.

## Results

Out of the 133 participants included in the analyses, 94 (71%) declared that they had consumed 7 drinks per week or fewer and 2 drinks per occasion or fewer in the past year, and 39 (29%) declared that they had exceeded one (N = 27) or both (N = 12) of these limits. They were respectively assigned to the **lower-risk** (N = 94, mean age = 68.79 (3.24), 64.9% women) and **higher-risk** (N = 94, mean age = 70.38 (4.73), 56.4% women) groups. In the overall sample, only two participants were abstinent from alcohol, including one lifelong abstainer and one former consumer of alcohol. These participants were included in the lower-risk group. Group comparisons were repeated without these two participants to ensure that they did not influence the results.

### Comparisons of alcohol consumption groups: higher-risk versus lower-risk

#### Demographics and potentially confounding variables

The higher-risk group consumed a mean of 9.1 ± 6.1 drinks per week, while the lower-risk group consumed a mean of 2.7 ± 2.3 drinks per week. Given the high heterogeneity in the higher-risk group, which included several heavy drinkers, all analyses were replicated excluding those excessive drinkers (defined as more than 14 drinks per week, *N = 5*). Smoking statuses were significantly different (*χ*^*2*^ = 7.27, *p* = 0.026, *V* = 0.23) with a higher proportion of former smokers in the higher-risk group and a higher proportion of never-smokers in the lower-risk group. The proportion of *APOE*4 carriers was significantly higher in the lower-risk group (*χ*^*2*^ = 4.24, *p* = 0.039, *V* = 0.18). Subsequent group comparisons were repeated with smoking status and *APOE*4 status as additional covariates. Otherwise, groups did not significantly differ on any demographic or potential confounding variables (Table 1, Supplementary Tables S1 and S2).

**Table 1:** Description of demographics and potentially confounding variables.

Variables	All *N* = 133	Lower-risk group *N* = 94	Higher-risk group *N* = 39	Comparisons
Demographics				
Age, years	69.26 (3.79) [65; 84]	68.79 (3.24) [65; 79]	70.38 (4.73) [65; 84]	*W* = 1505.50, *p* = 0.104, *r* = 0.14
Sex (%)—Women	83 (62.4%)	61 (64.9%)	22 (56.4%)	*χ*^*2*^ = 0.52, *p* = 0.47, *V* = 0.06
Men	50 (37.6%)	33 (35.1%)	17 (43.6%)	
Education, years	13.17 (3.09) [7; 22]	13.38 (3.18) [7; 22]	12.64 (2.84) [7; 17]	*W* = 1621.50, *p* = 0.291, *r* = 0.09
Alcohol consumption				
Drinks per week, number	4.57 (4.82) [0; 24.50]	2.70 (2.34) [0; 7]	9.09 (6.13) [0.47; 24.50]	*W* = 629.00, ***p ≤* 0.001∗∗∗,***r* = 0.52
Drinks per occasion, number	2.06 (1.18) [1; 7.50]	1.46 (0.50) [0; 2]	3.47 (1.09) [1.50; 7.50]	*W* = 168, ***p ≤* 0.001∗∗∗,***r* = 0.76
Genetic risk for Alzheimer’s disease				
*APOE*4 status—non-carriers	98 (73.7%)	64 (68.1%)	34 (87.2%)	*χ*^*2*^ = 4.24, ***p =* 0.039∗,** V = 0.18
Carriers	35 (26.3%)	30 (31.9%)	5 (12.8%)	
Medical treatments				
Cardiovascular treatments, number^a^	0.50 (0.76) [0; 3]	0.54 (0.81) [0; 3]	0.41 (0.59) [0; 2]	*W* = 1916.50, *p* = 0.631,*r* = 0.04
Anti-inflammatory treatments, number	0.02 (0.12) [0; 1]	0.01 (0.10) [0; 1]	0.03 (0.16) [0; 1]	*W* = 1805.50, *p* = 0.527, *r* = 0.06
Hormonal treatments for thyroid, number	0.16 (0.39) [0; 2]	0.16 (0.40) [0; 2]	0.15 (0.37) [0; 1]	*W* = 1827.00, *p* = 0.965, *r* = 0.00
Hormonal treatments for menopause, number	0.15 (0.50) [0; 2]	0.16 (0.51) [0; 2]	0.14 (0.47) [0; 2]	*W* = 697.00, *p* = 0.945, *r* = 0.01
Anxiety and depression				
STAI-B (/80)	34.58 (7.05) [20; 54]	34.78 (6.81) [50; 54]	34.10 (7.66) [20; 51]	*t* = −0.48, *p* = 0.635, *d* = −0.10
GDS (/30)	1.30 (1.75) [0; 11]	1.36 (1.83) [0; 11]	1.15 (1.53) [0; 6]	*W* = 1920.50, *p* = 0.65, *r* = 0.04
Lifestyle				
LEQ^b^, total score	97.90 (17.97) [56; 143]	98.49 (18.15) [56; 143]	96.49 (17.69) [59; 131]	*t* = −0.59, *p* = 0.557, *d* = 0.11
Smoker status—Current	7 (5.3%)	4 (4.3%)	3 (7.7%)	*χ*^*2*^ = 0.15, *p =* 0.703, *V =* 0.03
Former	61 (45.9%)	37 (39.4%)	24 (61.5%)	*χ*^*2*^ = 4.60, ***p =* 0.032∗,** *V =* 0.19

Data are N (%) or mean (sd) [range] for the whole sample and by groups of alcohol consumption. Groups were compared with chi2 (χ2) for qualitative variables and with Student's t-tests (t) or Wilcoxon rank sum tests (W), depending on normality of the distribution. Effect sizes are specified with Cramer's V, Cohen's d, or Wilcoxon's r. Significant comparisons are flagged in bold. ∗∗∗: *p* < 0.001; ∗: *p* < 0.05.

GDS, Geriatric Depression Scale^48^; STAI-B, State-Trait Anxiety Inventory B^49^; LEQ, Lifetime of Experiences Questionnaire.^50^

a Number of cardiovascular treatments is the total of either antihypertensive, anticoagulant/antiplatelet, diabetes treatment, hypoglycaemic treatment, and cholesterol-lowering treatment.

b The total LEQ score is reported in this table. Sub-scores by life periods are described in Supplementary Table S1.

#### Cognition

ANCOVAs revealed no significant difference in any of the composite cognitive scores (Table 2) nor any of the scores used to form the composites (Supplementary Table S3). Similar results were obtained in analyses excluding *N* = 2 abstainers or *N* = 5 excessive drinkers (Supplementary Tables S3 and S4), as well as in analyses including either *APOE*4 status or smoking status as additional covariates (Supplementary Tables S5 and S6). Given the absence of group differences, dose–response relationships were not investigated.

**Table 2:** Lower-risk versus higher-risk group comparisons for cognitive scores.

Variables	All *N* = 133	Lower-risk group *N* = 94	Higher-risk group *N* = 39	Comparisons
Episodic memory	0.02 (1.00) [−2.81; 1.93]	0.14 (0.98) [−2.49; 1.93]	−0.26 (0.98) [−2.81; 1.32]	*F* = 1.818, *p* = 0.180, *d =* 0.264
Executive functions	0.02 (0.99) [−3.04; 2.46]	0.08 (1.00) [−2.24; 2.46]	−0.11 (0.97) [−3.04; 2.24]	*F* = 0.005, *p* = 0.944, *d =* 0.014
Attention-processing speed	0.02 (0.99) [−1.87; 2.09]	0.07 (1.00) [−1.87; 2.09]	−0.09 (0.99) [−1.70; 2.03]	*F* = 0.069, *p* = 0.793, *d =* 0.052

Data are mean (sd) [range] of standardised composite z-scores, reported for the whole sample and for each group. Groups were compared with ANCOVAs controlling for age, sex, and level of education. Effect sizes are specified with Cohen's d.

#### Structural cerebral integrity

**Fig. 1:**
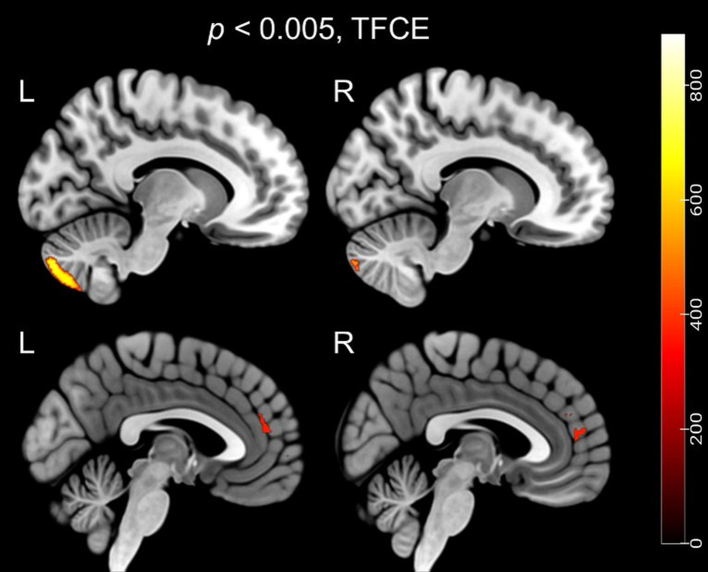
**Lower-risk versus higher-risk group comparisons for grey matter volume (N = 133)**. Grey matter volume was compared between groups in a *two-sample t-test* (SPM12) correcting for age, sex and level of education. Significant clusters (*p* < 0.005, TFCE) from the *higher-risk < lower-risk* contrast are projected into four sagittal slices. TFCE values are indicated on a red-yellow colour bar.

Regarding the grey matter analysis (Fig. 1), the higher-risk group had lower volume than the lower-risk group in cerebellar hemispheres, more precisely in the bilateral Crus II and the left cerebellar lobules VIIb and VIIIa, as well as in a small bilateral cluster located in the anterior cingulate (*p* < 0.005, TFCE). No between-group difference was observed with FWE comparisons.

**Fig. 2:**
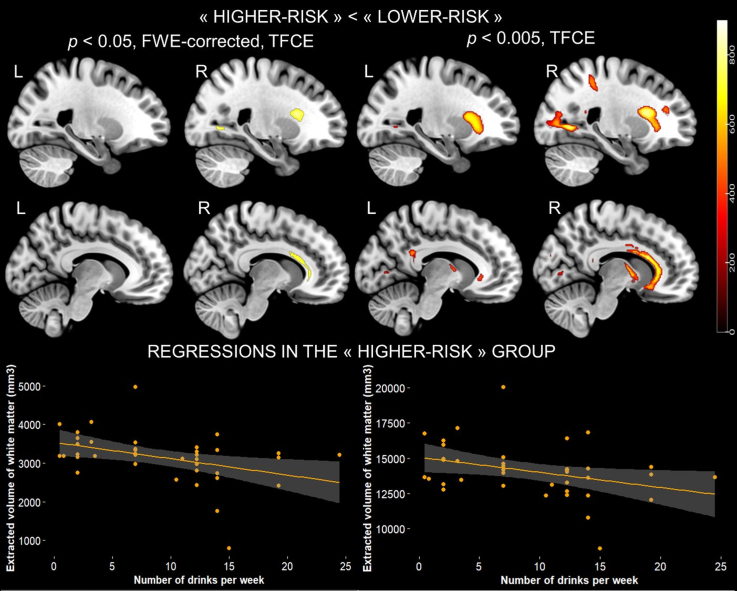
**Lower-risk versus higher-risk group comparisons for white matter volume and regression between volumes and number of drinks per week in the higher-risk group (N = 133)**. White matter volume was compared between groups in a *two-sample t-test* (SPM12) correcting for age, sex and level of education. Significant clusters from the *higher-risk < lower-risk* contrast are projected onto four sagittal slices for both FWE-corrected (*p* < 0.05, TFCE) and uncorrected results (*p* < 0.005, TFCE). TFCE values are indicated on a red-yellow colour bar. Regression between volumes and number of drinks per week is rendered below the corresponding representation of significant clusters from which volume was extracted (*left:* clusters from FWE-corrected results (*p* < 0.05, TFCE); *right*: clusters from uncorrected results (*p* < 0.005, TFCE)).

Regarding the white matter volume (Fig. 2), the higher-risk group showed widespread clusters of lower volume (*p* < 0.005, TFCE) in the corpus callosum, inferior longitudinal and fronto-occipital fasciculi, cingulum bundles, corticospinal tracts and in the internal capsules. Additional volume differences were observed in the right inferior longitudinal fasciculus, right optic radiations and bilateral fornix. Following correction for multiple comparisons (TFCE, FWE-corrected, *p* < 0.05), significant volume differences were localised predominantly in the white matter tracts of the right hemisphere, including the anterior and middle parts of the corpus callosum, the inferior longitudinal and fronto-occipital fasciculus, posterior thalamic radiations and the internal capsule.

**Fig. 3:**
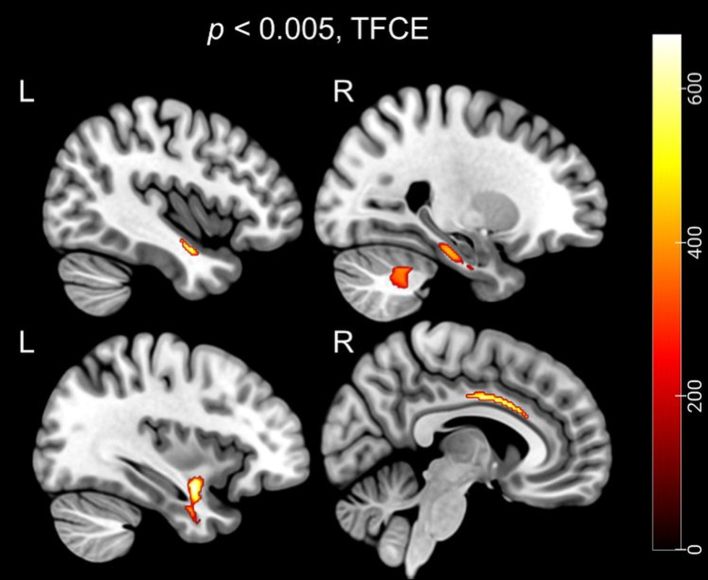
**Lower-risk versus higher-risk group comparisons for white matter microstructure integrity (N = 132)**. Mean diffusivity values were compared between groups in a *two-sample t-test* (SPM12) correcting for age, sex and level of education. Significant clusters (*p* < 0.005, TFCE) from the *lower-risk < higher-risk* contrast are projected into four sagittal slices. TFCE values are indicated on a red-yellow colour bar.

Regarding white matter microstructure integrity (Fig. 3), the higher-risk group displayed higher MD values in clusters located in the left cerebellar hemisphere/middle cerebellar peduncle, temporal regions of white matter and in the right cingulum (*p* < 0.005; TFCE), but these differences did not survive corrections for multiple comparisons. In addition, no significant group differences were observed using FA values.

For both grey and white matter, similar results were obtained in analyses excluding N = 2 abstainers (Supplementary Figures S2–S4), as well as in analyses including APOE4 status as an additional covariate (Supplementary Figures S10–S12). The same clusters were also obtained in analyses including smoking status as an additional covariate, but of considerably smaller size (Supplementary Figures S13–S15). In analyses excluding excessive drinkers (*N* = 5), similar results were obtained for grey matter (Supplementary Figure S6) and for MD values in white matter (Supplementary Figure S8), while differences in white matter volume no longer survived multiple comparison correction (Supplementary Figure S7).

#### Amyloid burden

**Fig. 4:**
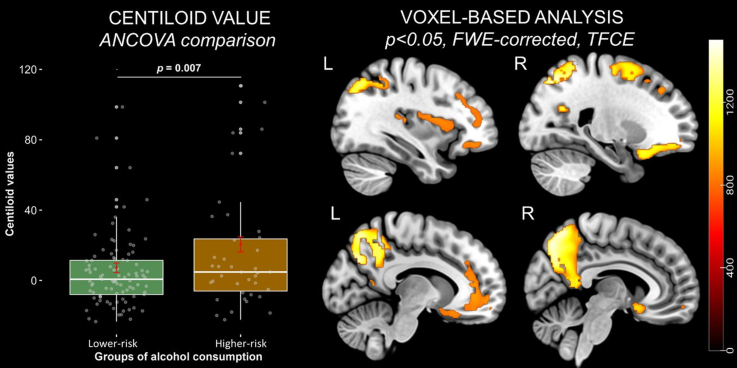
**Lower-risk versus higher-risk group comparisons for amyloid load (N = 132)**. **Left:** Boxplots illustrate the distribution of Centiloid values within each group of alcohol consumption. Individual data points are shown as jittered dots to display the distribution. Estimated marginal means and standard errors are in red. Groups were compared on a Type III ANCOVA correcting for age, sex, level of education and *APOE*4 status. **Right:** Mean AV45 SUVr values were compared between groups in a *two-sample t-test* (SPM12) correcting for age, sex, level of education and *APOE*4 status. Significant clusters from the *lower-risk < higher-risk* contrast are projected onto four sagittal slices for FWE-corrected results (*p* < 0.05, TFCE). TFCE values are indicated on a red-yellow colour bar.

The ANCOVA model showed that Centiloid values were significantly higher in the higher-risk group (15.24 ± 33.57) than in the lower-risk group (4.41 ± 20.19) (*F* = 7.577; *p* = 0.007; *d =* 0.553) (Fig. 4). The same results were obtained with the permutation ANOVA (*F* = 7.577; *p* = 0.007; *d =* 0.553).

In the voxel-wise analysis, the higher-risk group showed significantly more amyloid deposition in widespread cortical regions (TFCE, FWE-corrected, *p* < 0.05) (Fig. 4). The most prominent cluster was located bilaterally in the temporo-parietal regions, including the precuneus, inferior and superior parietal lobules, angular and supramarginal gyri, middle and superior temporal gyri, and the right inferior temporal gyrus. In the higher-risk group, more amyloid deposition was also found bilaterally in the frontal regions, including the gyrus rectus, superior orbital frontal gyrus, orbital part of the middle frontal gyrus, as well as the inferior and superior frontal gyri. More amyloid deposition in the higher-risk group was also located in the bilateral insula and Rolandic operculum.

Similar results were obtained in analyses excluding *N* = 2 abstainers or *N* = 5 excessive drinkers (Supplementary Figures S5–S9), as well as in analyses including smoking status as an additional covariate (Supplementary Figure S16).

### Dose-dependent links between brain alterations and alcohol consumption in the higher-risk group

In the higher-risk group, total white matter volume (mm^3^) extracted from significant clusters of the two-sample contrast higher-risk < lower-risk was negatively associated with the number of drinks per week (Fig. 2). In the total volume of clusters identified in the analysis corrected for multiple comparisons (*p* < 0.05, FWE-corrected), an increase of one drink per week was associated with a decrease of 38.64 mm^3^ (*β* = −38.64; 95% CI [−70.847; −6.430]; *p* = 0.020; *R2* = 0.515). In the total volume of clusters identified in the uncorrected analysis (*p* < 0.005, TFCE), an increase of one drink per week was associated with a decrease of 92.26 mm^3^ (*β* = −92.26; 95% CI [−171.177; −13.341]; *p* = 0.023; *R2* = 0.174). No significant association was observed between extracted grey matter volume, mean Florbetapir SUVr values or Centiloid value (all *p* values > 0.10).

## Discussion

This study aimed to compare various markers of neurocognitive health in cognitively healthy older adults based on whether they complied or not with recent French-specific alcohol consumption recommendations for elderly subjects (lower-risk *versus* higher-risk groups). Despite no difference in measures of cognitive performance, the higher-risk group presented with lower cerebral integrity. The most significant result was elevated amyloid burden in the higher-risk group, a biomarker associated with a higher risk of Alzheimer's disease–related cognitive decline and dementia. In addition, brain volume was lower in the higher-risk group in cerebellar and anterior cingulate grey matter, as well as in widespread white matter regions. Regarding white matter microstructural integrity, higher mean diffusivity values were observed in the left cerebellum, the right cingulum, and in temporal regions in the higher-risk group, while groups did not differ in terms of fractional anisotropy. A dose–response relationship between significantly different white matter volume and the number of drinks per week was also observed in the higher-risk group.

A study, based on the French cohort Constances^51^ involving more than thirty thousand respondents, showed that 36% of women exceeded recommended drinking guidelines, all ages combined, while 67% of men aged 64–73 exceeded these guidelines, significantly more than the 20–34 age group (59%).^52^ As less than 30% of our participants consumed above low-risk alcohol consumption thresholds, our findings may underestimate the detrimental effects associated with higher-risk consumption in the national senior population. Still, our study offers one of the few neuroimaging evidences of an elevated cerebral amyloid burden associated with alcohol consumption in a healthy population. This finding contrasts with previous studies reporting either no amyloid association with current alcohol consumption in healthy older adults^53^^,^^54^ or associations between moderate lifetime alcohol and lower amyloid deposition compared to abstinence.^23^ We hypothesise that we were able to observe more amyloid deposition in the higher-risk group thanks to comparability between groups in a wide range of variables, apart from the ones included in sensitivity analyses. We found no linear dose–response relationship with the number of drinks per week in this group, suggesting that this indicator of alcohol consumption may not be the right one to reflect the association between alcohol and amyloid accumulation. Neuroinflammation could explain an alcohol-related increase in amyloid accumulation, as proposed in patients with alcohol use disorder.^55^ Alcohol-related neuroinflammation could impair amyloid clearance via dysregulated microglial function.^55^^,^^56^ Overall, our associative results from an observational study are in line with previous studies using Mendelian randomisation, which suggested a causal increased risk of dementia,^18^ especially Alzheimer's disease,^57^ even with low-to-moderate alcohol consumption.

We also observed altered structural brain integrity in the higher-risk group, most significant and widespread for white matter volume. Group differences were notably located in the corpus callosum, cingulum, and tracts connecting temporal and occipital regions. In these regions, a dose–response association was observed in the higher-risk group. This association needs to be interpreted cautiously due to the low sample size in this group. The group difference in white matter volume appeared less widespread when excessive drinkers were excluded, highlighting the vulnerability of white matter alterations to heavy drinking. This is consistent with previous results reporting negative associations between alcohol consumption and white matter macrostructure in the general population,^16^ as well as with the robust literature on alcohol use disorder.^58^ White matter is altered by excessive alcohol consumption, which notably provokes demyelination and axonal loss.^59^^,^^60^ The corpus callosum could be particularly vulnerable to alcohol since it is early and significantly affected in alcohol use disorder.^58^

While amyloid burden and white matter volume seem sensitive to differentiate our groups, results for white matter microstructural integrity or grey matter volume did not survive corrections for multiple comparisons. Previous studies have reported a negative association between alcohol consumption and grey matter volume or white matter microstructural integrity affecting a range of brain regions.^14^^,^^16^ Herein, our groups differ in terms of microstructural integrity in mean diffusivity in the cerebellum, cingulum and in temporal regions, without any group difference in terms of fractional anisotropy. We found lower grey matter volume in the higher-risk group in selective regions only, including the cerebellum and the anterior cingulate cortex. These regions are involved in motor and executive functions, which have been previously described as being altered in patients with alcohol use disorder.^61^^,^^62^ While executive functioning was also shown to be negatively associated with alcohol consumption in the general population,^15^ in the present study, there were no differences in cognition (i.e., executive functioning, attention-processing speed or verbal episodic memory). This could be explained by the high level of education of our sample. Higher cognitive reserve in older adults with risky alcohol consumption and associated cerebral alterations could delay the onset of cognitive symptoms. Previous studies have observed a negative association between cognition and alcohol consumption only in participants with a low level of education.^14^ A longitudinal follow-up may reveal that prolonged risky alcohol consumption is associated with more severe cognitive decline. Still, observed amyloid accumulation in the absence of group differences in cognition is consistent with the progression of Alzheimer's disease since amyloid accumulation begins years before the onset of functional symptoms.^63^ Increased amyloid accumulation in cognitively healthy older adults is a risk factor for Alzheimer's disease–related cognitive decline and dementia.^64^, ^65^, ^66^, ^67^ Our sample of higher-risk drinkers thus appears at such a higher risk.

Finally, in both grey matter and white matter comparisons, clusters indicating lower volume in the higher-risk group were more limited in analyses correcting for smoking status, despite being localised in the same regions. This attenuation could reflect an additive effect of alcohol and smoking on cerebral structural integrity.^34^^(preprint)^ However, our ability to interpret the potential interactive effects of alcohol and smoking is limited by differences in the definition of our variables, namely, lifetime smoking *versus* past-year alcohol consumption. Future studies should explore the impact of current and past alcohol and smoking on the neurocognitive health of older people, using harmonised measures of consumption of these two substances.

This study has several limitations. First, its observational cross-sectional design does not allow for conclusions about causality or temporal relationships. Even if we could compare our groups on a wide range of variables, residual confounding may still play a role in the observed associations. For example, personality traits (i.e., neuroticism) could influence both alcohol consumption^68^ and neurocognitive integrity.^69^ In addition, reverse causation cannot be excluded in our observational study. Second, as in all clinical studies including alcohol measures, data reflect self-reported patterns of alcohol consumption, which may be subject to recall or social desirability biases, potentially affecting the accuracy of the reported levels. The use of objective markers of alcohol consumption (e.g., phosphatidylethanol) is a promising outlook in alcohol research. Third, while our study has the strength to use a classification of higher-risk drinking based on both weekly and occasion-based drinking limits, a more comprehensive investigation of drinking patterns would be relevant for future studies (including binge drinking episodes or subgroups depending on compliance with weekly-based, occasion-based or both thresholds) and could further refine our understanding of drinking behaviours and their potential implications. Fourth, the relatively small sample size, particularly in the higher-risk drinking group, limits the statistical power to detect small-to-moderate effect sizes and reduces the precision of effect estimates. Larger samples will be needed to replicate and extend our findings, notably to investigate sex-specific associations. Fifth, as participants were recruited from a community-based RCT, the cohort may not be fully representative of the general older adult population, with likely healthier and more educated participants, which may limit the generalisability of our findings. The proportion of higher-risk drinkers in our sample is lower than national estimates, suggesting our findings may underestimate the detrimental effects of alcohol on brain integrity at a population level.

This study investigates differences in neurocognitive health among cognitively healthy older adults based on compliance with French alcohol consumption safety guidelines designed for older adults. Despite no group differences in cognitive performance, individuals who did not follow the guidelines showed higher amyloid burden and worse structural brain integrity, even for moderate levels of alcohol consumption. Further longitudinal research is needed to determine whether these neurocognitive differences contribute to cognitive decline or the development of neurodegenerative diseases such as Alzheimer's disease. It is important to highlight the fact that recommendations define the threshold of low-risk consumption and not the threshold of safe consumption, as it is underlined by the growing evidence that no amount of alcohol can be considered entirely safe. Findings support stricter adherence to alcohol guidelines in older adults, but more globally, better communication about age-related increased risk to alcohol consumption, as well as a reconsideration of public health recommendations for older adults, in France and on the international scene.

## Contributors

Concept and design: CS, SS, ALP. Acquisition, analysis, or interpretation of data: CS, JG, RdF, BL, FM, GC, SS, ALP. Drafting of the manuscript: CS, SS, ALP, GC. Critical revision of the manuscript for important intellectual content: CS, JG, NC, AL, NM, FC, RdF, EF, BL, FM, GC, SS, ALP. Statistical analysis: CS. Obtained funding: GC, NM, FC. Administrative, technical, or material support: RdF, BL, FM. Supervision: SS, ALP. Other–Principal investigators: GC. The Medit-Ageing Research Group conceptualised the Age-Well clinical Trial and collected data. CS and ALP have accessed and verified the underlying data. All authors have read and approved the final version of the manuscript.

## Data sharing statement

The data underlying this manuscript are made available on request following a formal data sharing agreement and approval by the consortium and executive committee (https://silversantestudy.eu/2020/09/25/data-sharing). The Material can be mobilised, under the conditions and modalities defined in the Medit-Ageing Charter, by any research team belonging to an Academic for carrying out a scientific research project relating to the scientific theme of mental health and well-being in older people. The Material may also be mobilised by non-academic third parties, under conditions, in particular financial, which will be established by a separate agreement between Inserm and the said third party. Data sharing policies described in the Medit-Ageing charter are in compliance with our ethics approval and guidelines from our funding body.

## Declaration of interests

JD received travel support for the Alzheimer Association International Conference 2024. All other authors declare no competing interests.

## Appendix A Supplementary data

Supplementary Materials

Collab group
