# Supplementary material for: Higher amyloid deposition and lower white matter volume in cognitively healthy seniors exceeding French alcohol recommendations
Source: eBioMedicine. 2026 Jul 15;130:106376. doi: 10.1016/j.ebiom.2026.106376 (PMC13382593; doi:10.1016/j.ebiom.2026.106376)
Supplement: Supplementary Materials [file mmc1.pdf]

Supplementary materials

1 Supplementary method 2
1.1 Inclusion and exclusion criteria 2
1.1.1 Inclusion Criteria 2
1.1.2 Exclusion Criteria 2
1.2 Flow chart 4
1.3 Centiloid value 5
2 Supplementary results 6
2.1 Detailed scores for potentially cofounding variables 6
2.2 Detailed scores for neuropsychological evaluation 7
2.3 Replication without non-drinkers 8
2.3.1 Cognition 8
2.3.2 Structural integrity 9
2.3.3 Amyloid burden 11
2.4 Replication without excessive-drinkers 12
2.4.1 Cognition 12
2.4.2 Structural integrity 13
2.4.3 Amyloid burden 15
2.5 Replication with APOE4 status as additional covariates 16
2.5.1 Cognition 16
2.5.2 Structural integrity 17
2.6 Replication with smoker status as additional covariates 19
2.6.1 Cognition 19
2.6.2 Structural integrity 20
2.6.3 Amyloid burden 22
2.7 References 23

1 Supplementary method

1.1 Inclusion and exclusion criteria

1.1.1 Inclusion Criteria

- Age ≥ 65 years
- Autonomous
- Living at home
- Educational level ≥ 7 years (from the Preparatory Course - 1st grade - included)
- Registered with the social security system
- Motivated to effectively participate in the project and signed the informed consent form
- Performance within the normal range on standardised cognitive tests according to agreed study specific standards (age, sex and level of education level when available)
- Native French speaker - Available to attend the intervention for the trial duration (24 months)
- Retired for at least one year
- No strong preference or aversion for an intervention group
- No present or past regular or intensive practice of meditation or comparable practices; practice is considered regular and/or intensive if it occurred more than one day per week for more than six consecutive months over the last 10 years, and/or in case of more than five consecutive days of intensive practice (internship or retreat) over the past 10 years, and/or of more than 25 days of retreats (cumulatively) within the last 10 years
- Not fluent in English

1.1.2 Exclusion Criteria

- Safety concerns related to MR scanning (claustrophobia, presence of ferromagnetic objects) or PET scanning (blood sampling to check hepatic and renal functions are performed before the PET scans; known hypersensibility to Amyvid® or GlucoPET®)
- Presence of a major neurological or psychiatric disorder (including addiction to alcohol or drugs)

- History of cerebral disease (vascular, degenerative, physical malformation, tumour, or head trauma resulting in loss of consciousness for more than an hour)
- Presence of a chronic disease or acute unstable illness (respiratory, cardiovascular, digestive, renal, metabolic, hematologic, endocrine or infectious)
- Current or recent use of medication that may interfere with cognitive functioning (psychotropic drugs, antihistamines with anticholinergic action, anti-Parkinson's medications, benzodiazepines, long-term steroidal anti-inflammatory treatment, antiepileptic drugs or analgesics), the interfering nature of different treatments is at the discretion of the investigating doctor
- Being under legal guardianship or incapacitation
- Participation in another biomedical research protocol involving the injection of radiopharmaceuticals
- Physical or behavioural inability to attend follow-up visits as planned in the study protocol

1.2 Flow chart

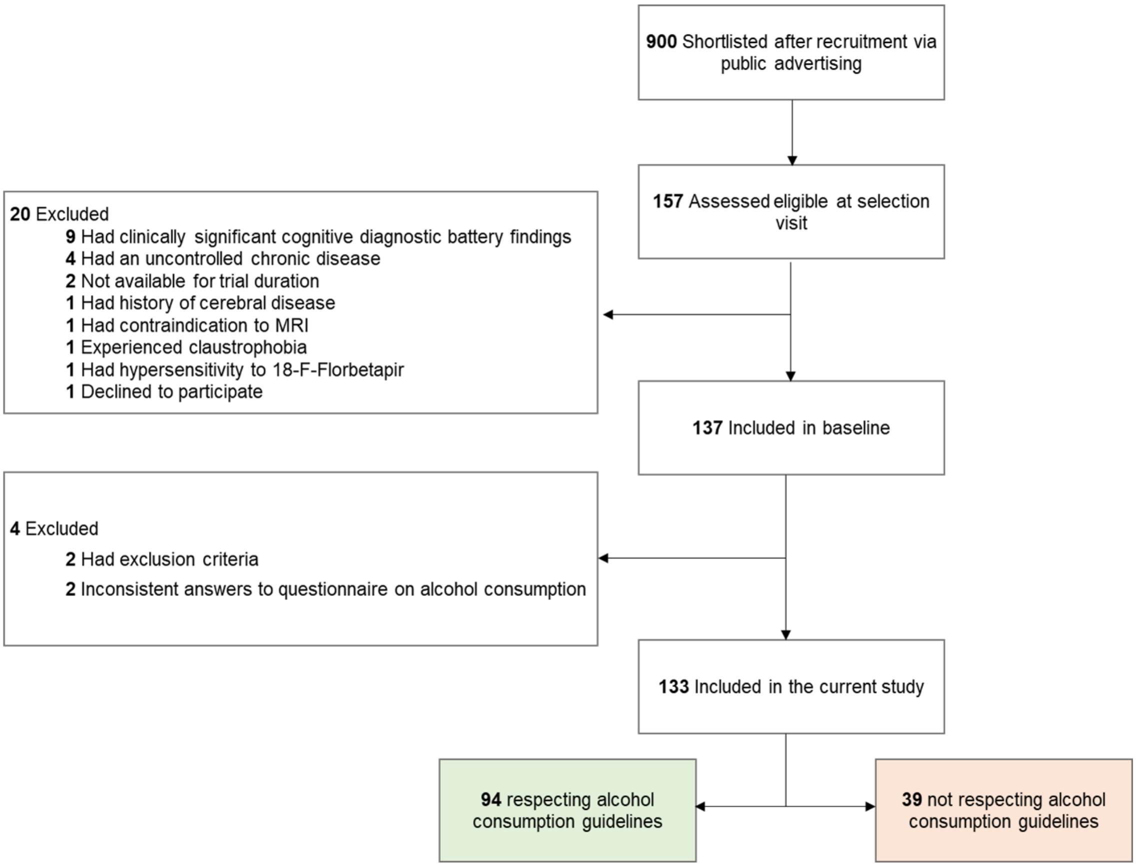

Supplementary Figure 1 Flow diagram of the study

1.3 Centiloid value

Amyloid uptake was extracted and averaged across AD-sensitive regions, including the frontal, temporal and parietal cortices, the precuneus, the anterior striatum and the insular cortex and SUVRs were transformed to Centiloid values by applying the standardised procedure proposed by Klunk et al (2015). For validation steps, we first assessed the zero-to-100 Centiloid scale from the same data as theirs (PiB-PET data from GAAIN) and confirmed that our results matched theirs, demonstrating our ability to reproduce their findings. We also preprocessed their data with our pipeline to assess any variations in results. Our in-home preprocessing yielded similar outcomes, meeting validation criteria. Given that Klunk and collaborators' procedure was designed for the PiB tracer, while our study employed Florbetapir, we applied the conversion procedure proposed by Navitsky and collaborators using their data, which encompassed both PiB- and Florbetapir-PET imaging in the same individuals (Navitsky et al., 2018). We derived the zero-to-100 Centiloid scale in PiB-PET data and then applied the conversion formula to translate Florbetapir SUVRs from PET volumes of interest into the Centiloid scale. The correlation between PiB- and Florbetapir-derived Centiloid values satisfied validation criteria.

2 Supplementary results

2.1 Detailed scores for potentially cofounding variables

	**ALL** *N*=133	**« Lower-risk » group** *N*=94	**« Higher-risk » group** *N*=39	**Comparisons**
LEQ young adulthood	31.42 (9.33) [10.00;52.83]	31.50 (9.30) [10.00;52.83]	31.21 (9.52) [11.20;52.40]	*t*=-0.16, *p*=0.872, *d*=0.03
LEQ midlife	38.37 (8.53) [19.75;65.65]	38.71 (9.16) [19.75;65.65]	37.55 (6.80) [24.25;51.90]	*t*=-0.81, *p*=0.422, *d*=0.14
LEQ late-life	28.12 (4.51) [17.40;38.40]	28.28 (4.27) [18.00;38.40]	27.73 (5.09) [17.40;37.80]	*t*=-0.59, *p*=0.557, *d*=0.12

Supplementary Table 1 Group description and comparison for life period LEQ subscores. Data are N (%) or mean (sd) [range] for the whole sample and by groups of alcohol consumption. Groups were compared with Student's t-tests (*t*). Effect sizes are specified with Cohen's *d*.
LEQ: Lifetime of Experiences Questionnaires

2.2 Detailed scores for neuropsychological evaluation

	Variables	ALL *N*=133	« Lower-risk » group *N*=94	« Higher-risk » group *N*=39	Comparisons
Episodic Memory	**CVLT-II** – sum of trials 1-5, *score*	0.03 (0.99) [-3.41;1.91]	-0.29 (1.11) [-3.41;1.42]	0.15 (0.90) [-2.30;1.91]	*F*=2.725 ; *p*=0.101 ; *d*=0.323
	– immediate free recall, *score*	0.03 (0.98) [-2.58;1.49]	-0.23 (1.11) [-2.58;1.49]	0.14 (0.90) [-2.21;1.49]	*F*=1,514 ; *p*=0.221 ; *d*=0.241
	– delayed free recall, *score*	0.02 (0.99) [-3.87;1.28]	-0.19 (1.08) [-3.87;1.28]	0.11 (0.93) [-3.13;1.28]	*F*=0,581 ; *p*=0.447 ; *d*=0.149
	**Logical Memory, Story B** – immediate free recall, *score*	-0.00 (1.01) [-3.15;2.44]	-0.15 (1.01) [-3.15;1.46]	0.06 (1.01) [-1.83;2.44]	*F*=0,581 ; *p*=0.447 ; *d*=0.149
	– delayed free recall, *score*	0.00 (1.01) [-3.29;2.48]	-0.16 (1.01) [-3.29;1.91]	0.07 (1.01) [-2.13;2.48]	*F*=0.475 ; *p*=0.492 ; *d*=0.135
Executive functioning	**Digit span**- backward, *score*	0.01 (1.00) [-1.65;3.94]	-0.08 (0.93) [-1.15;3.43]	0.05 (1.04) [-1.65;3.94]	*F* < 0.001 ; *p*=0.981 ; *d*=0.005
	**Trail Making Test** – Part B, *response time*	0.01 (1.00) [-4.74;1.48]	-0.01 (1.05) [-4.74;1.17]	0.03 (0.98) [-3.83;1.48]	*F*=0.909 ; *p*=0.342 ; *d*=0.187
	**Stroop** – Interference index, *response time*	0.03 (0.97) [-4.53;1.77]	-0.04 (0.92) [-3.06;1.30]	0.05 (0.99) [-4.53;1.77]	*F*=0.157 ; *p*=0.692 ; *d*=0.078
	**Letter Fluency**, *score*	0.01 (1.00) [-2.82;2.89]	-0.19 (1.03) [-2.82;2.73]	0.09 (0.98) [-2.34;2.89]	*F*=1.156 ; *p*=0.284 ; *d*=0.211
Attention-Processing speed	**Trail Making Test** – Part A, *response time*	0.02 (1.00) [-3.01;1.74]	-0.04 (1.00) [-2.39;1.48]	0.04 (1.00) [-3.01;1.74]	*F*=0.076 ; *p*=0.783 ; *d*=0.054
	**Stroop** – Naming, *response time*	0.01 (1.00) [-4.90;2.30]	0.02 (0.99) [-1.77;1.89]	0.01 (1.01) [-4.90;2.30]	*F*=0.139 ; *p*=0.709 ; *d*=0.073
	**Digit span** – forward, *score*	0.01 (1.00) [-1.65;2.57]	-0.00 (0.95) [-1.65;2.57]	0.01 (1.03) [-1.65;2.57]	*F*=0.274 ; *p*=0.603 ; *d*=0.102
	**Coding**, *score*	0.02 (0.99) [-2.02;3.05]	-0.21 (1.01) [-2.02;2.16]	0.12 (0.97) [-1.86;3.05]	*F*=0.333 ; *p*=0.565 ; *d*=0.113

Supplementary Table 2 Results of ANCOVAs comparisons for scores included in the composite cognitive scores. Data are mean (sd) [range] of standardised scores, reported for the whole sample and for each group. Groups were compared with ANCOVAs controlling for age, sex and level of education. Effect sizes are specified Cohen's d. CVLT: California Verbal Learning Test.

2.3 Replication without non-drinkers

2.3.1 Cognition

	**ALL** **N=131**	**« Lower-risk » group** **N=92**	**« Higher-risk » group** **N=39**	**Comparisons**
**Episodic memory**	0.02 (1.00) [-2.81;1.93]	0.16 (0.98) [-2.49;1.93]	-0.26 (0.98) [-2.81;1.32]	F=2.093; p=0.150; d=0.284
**Executive functioning**	0.02 (0.99) [-3.04;2.46]	0.08 (1.00) [-2.24;2.46]	-0.11 (0.97) [-3.04;2.24]	F < 0.001; p=0.999; d<0.001
**Attention-processing speed**	0.02 (0.99) [-1.87;2.09]	0.07 (1.01) [-1.87;2.09]	-0.09 (0.99) [-1.70;2.03]	F=0.056; p=0.813; d=0.047

Supplementary Table 3 Results of ANCOVAs comparisons for cognitive scores without the participants who did not drink last year (n=2). Data are mean (sd) [range] of standardised composite scores, reported for the whole sample and for each group. Groups were compared with ANCOVAs controlling for age, sex and level of education. Effect sizes are specified with Cohen’s d.

2.3.2 Structural integrity

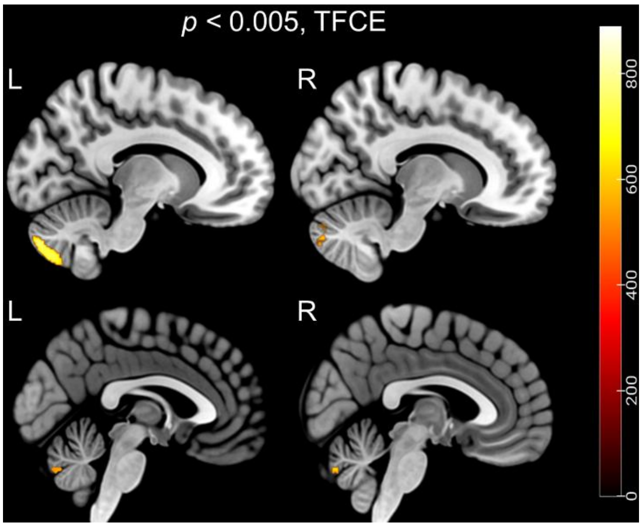

Supplementary Figure 2 Results for grey matter volume without the participants who did not drink last year (*N*=2). Grey matter volume was compared between groups in a two-sample t-test (SPM12) correcting for age, sex and level of education. Significant clusters (p<0.005, TFCE) from the *"higher-risk"* < *"lower-risk"* contrast are projected onto four sagittal slices. TFCE values are indicated on a red-yellow colour bar.

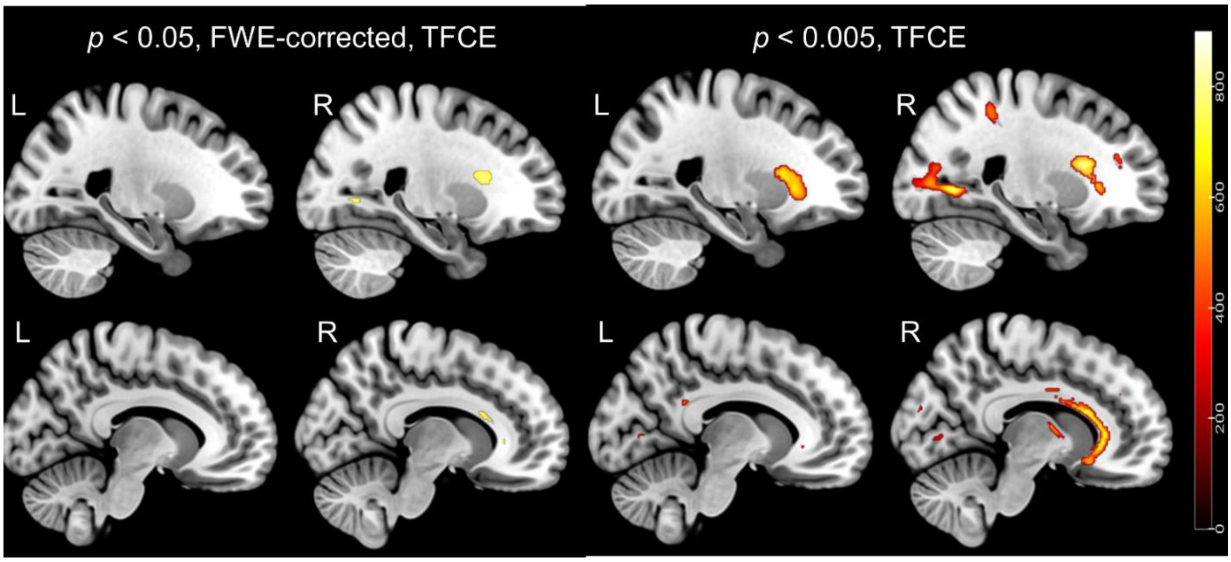

Supplementary Figure 3 Results for white matter volume without the participants who did not drink last year (*N*=2). White matter volume was compared between groups in a two-sample t-test (SPM12) correcting for age, sex and level of education. Significant clusters from the *"higher-risk"* < *"lower-risk"* contrast are projected onto four sagittal slices for both FWE-corrected (p<0.05, TFCE) and uncorrected results (p<0.005, TFCE). TFCE values are indicated on a red-yellow colour bar.

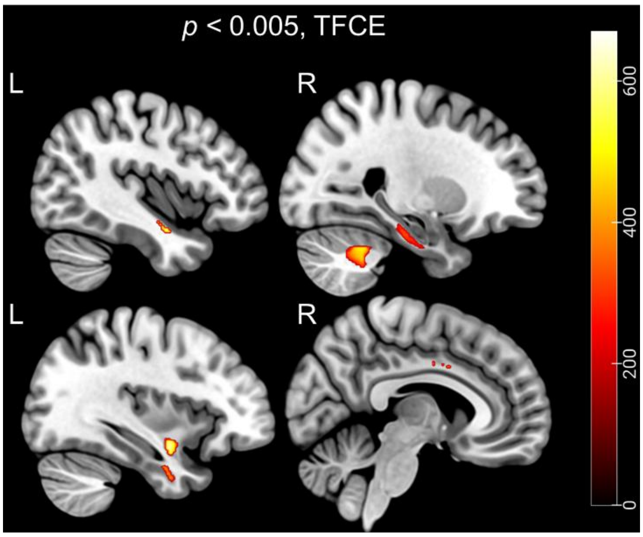

Supplementary Figure 4 Results for mean diffusivity values without the participants who did not drink last year (*N*=2).

Mean diffusivity values were compared between groups in a *two-sample t-test* (SPM12) correcting for age, sex and level of education. Significant clusters (*p*<0.005, TFCE) from the "*lower-risk*" < *"higher-risk*" contrast are projected into four sagittal slices. TFCE values are indicated on a red-yellow colour bar.

2.3.3 Amyloid burden

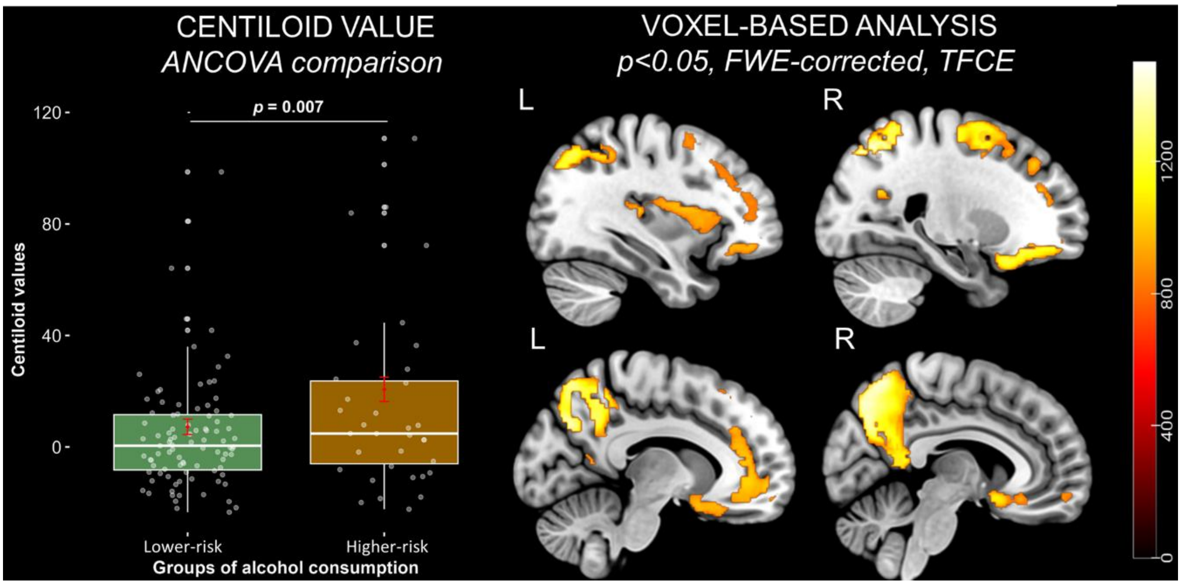

Supplementary Figure 5 Results for late AV45 SUVr without the participants who did not drink last year (*N*=2). Left: Boxplots illustrate the distribution of Centiloid values within each group of alcohol consumption. Individual data points are shown as jittered dots to display the distribution. Estimated marginal means and standard errors are in red. Groups were compared on a Type III ANCOVA correcting for age, sex, level of education and APOE status. **Right:** Mean AV45 SUVr values were compared between groups in a *two-sample t-test* (SPM12) correcting for age, sex, level of education and APOE status. Significant clusters from the "*lower-risk*" < "*higher-risk*" contrast are projected onto four sagittal slices for FWE-corrected ($p<0.05$, TFCE). TFCE values are indicated on a red-yellow colour bar.

2.4 *Replication without excessive-drinkers*

2.4.1 Cognition

	**ALL** ***N*=131**	**« Lower-risk » group** ***N*=94**	**« Higher-risk » group** ***N*=34**	**Comparisons**
**Episodic memory**	0.04 (0.97) [-2.49;1.93]	0.14 (0.98) [-2.49;1.93]	-0.22 (0.88) [-2.24;1.21]	*F*=1.683; *p*=0.197; *d*=0.264
**Executive functioning**	0.02 (0.99) [-3.04;2.46]	0.08 (1.00) [-2.24;2.46]	-0.15 (0.94) [-3.04;2.24]	*F*=0.077; *p*=0.782; *d*=0.056
**Attention-processing speed**	0.02 (0.98) [-1.87;2.09]	0.07 (1.00) [-1.87;2.09]	-0.12 (0.92) [-1.70;1.81]	*F*=0.026; *p*=0.873; *d*=0.033

Supplementary Table 4 Results of ANCOVAs comparisons for cognitive scores without the excessive drinkers (*N*=5). Data are mean (sd) [range] of standardised composite scores, reported for the whole sample and for each group. Groups were compared with ANCOVAs controlling for age, sex and level of education. Effect sizes are specified with Cohen's d.

2.4.2 Structural integrity

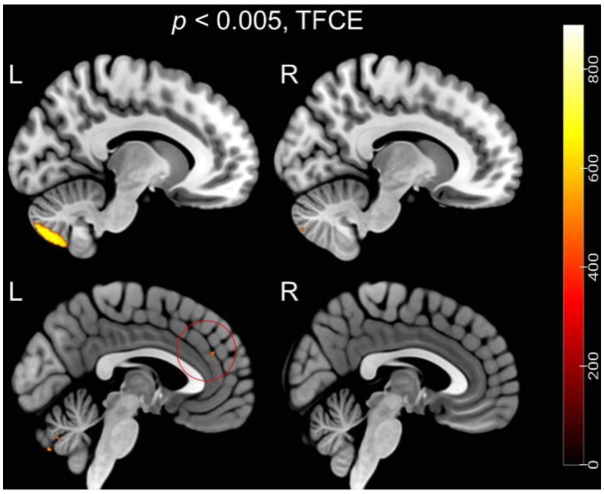

Supplementary Figure 6 Results for grey matter volume without the excessive drinkers (*N*=5). Grey matter volume was compared between groups in a two-sample t-test (SPM12) correcting for age, sex and level of education. Significant clusters (p<0.005, TFCE) from the *"higher-risk"* < *"lower-risk"* contrast are projected onto four sagittal slices. TFCE values are indicated on a red-yellow colour bar.

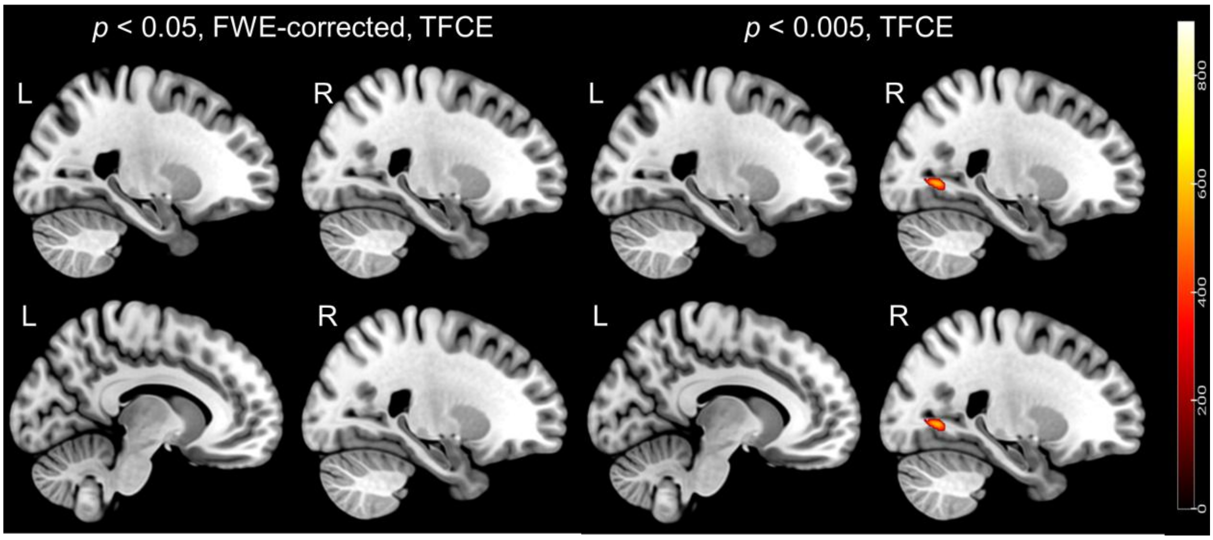

Supplementary Figure 7 Results for white matter volume without the excessive drinkers (*N*=5) White matter volume was compared between groups in a two-sample t-test (SPM12), correcting for age, sex and level of education. Significant clusters from the *"higher-risk"* < *"lower-risk"* contrast are projected onto four sagittal slices for both FWE-corrected (p<0.05, TFCE) and uncorrected results (p<0.005, TFCE). TFCE values are indicated on a red-yellow colour bar.

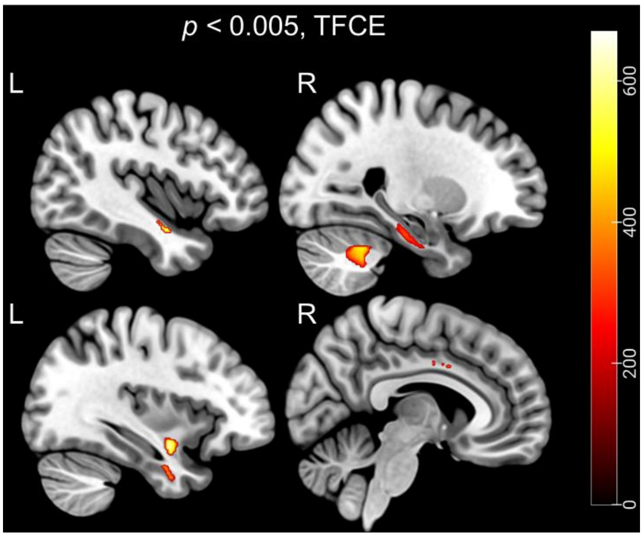

Supplementary Figure 8 Results for mean diffusivity values without the excessive drinkers (*N*=5). Mean diffusivity values were compared between groups in a *two-sample t-test* (SPM12) correcting for age, sex and level of education. Significant clusters (*p*<0.005, TFCE) from the "*lower-risk" < "higher-risk*" contrast are projected into four sagittal slices. TFCE values are indicated on a red-yellow colour bar.

2.4.3 Amyloid burden

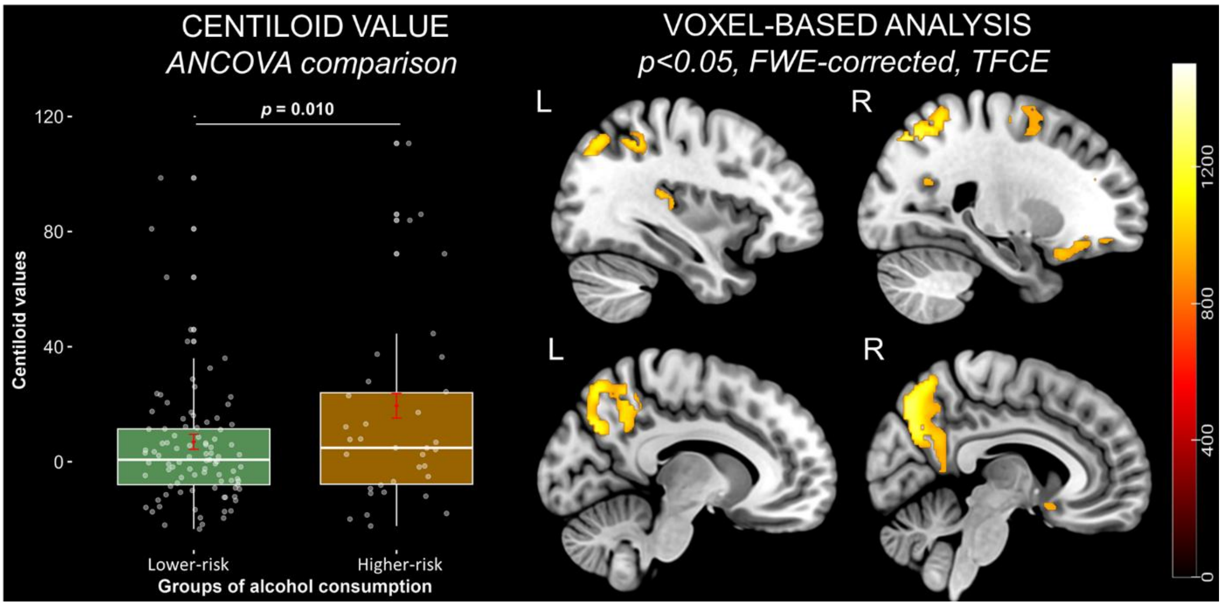

Supplementary Figure 9 Results for late AV45 SUVr without the excessive drinkers (*N*=5). Left: Boxplots illustrate the distribution of Centiloid values within each group of alcohol consumption. Individual data points are shown as jittered dots to display the distribution. Estimated marginal means and standard errors are in red. Groups were compared on a Type III ANCOVA correcting for age, sex, level of education and APOE status. **Right:** Mean AV45 SUVr values were compared between groups in a *two-sample t-test* (SPM12) correcting for age, sex, level of education and APOE status. Significant clusters from the "*lower-risk*" < *"higher-risk*" contrast are projected onto four sagittal slices for FWE-corrected (p<0.05, TFCE). TFCE values are indicated on a red-yellow colour bar.

2.5 Replication with APOE4 status as additional covariates

2.5.1 Cognition

	ALL *N*=131	« Lower-risk » group *N*=92	« Higher-risk » group *N*=39	Comparisons
**Episodic memory**	0.02 (1.00) [-2.81;1.93]	0.16 (0.98) [-2.49;1.93]	-0.26 (0.98) [-2.81;1.32]	*F*=1.435; *p*=0.233; *d*=0.240
**Executive functioning**	0.02 (0.99) [-3.04;2.46]	0.08 (1.00) [-2.24;2.46]	-0.11 (0.97) [-3.04;2.24]	*F*=0.091; *p*=0.764; *d*=0.060
**Attention-processing speed**	0.02 (0.99) [-1.87;2.09]	0.07 (1.01) [-1.87;2.09]	-0.09 (0.99) [-1.70;2.03]	*F*=0,138; *p*=0.710; *d*=0.074

Supplementary Table 5 Results of ANCOVAs comparisons for cognitive scores with APOE status as additional covariate. Data are mean (sd) [range] of standardised composite scores, reported for the whole sample and for each group. Groups were compared with ANCOVAs controlling for age, sex, level of education and APOE status. Effect size are specified with Cohen's d.

2.5.2 Structural integrity

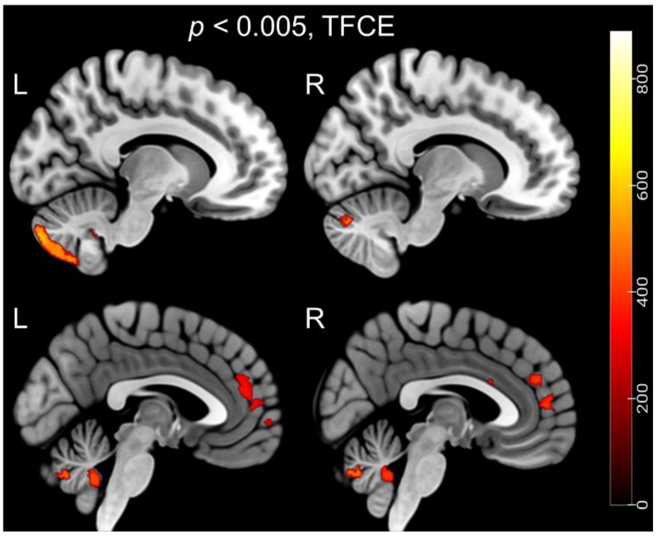

Supplementary Figure 10 Results for grey matter volume with APOE status as additional covariate. Grey matter volume was compared between groups in a *two-sample t-test* (SPM12) correcting for age, sex, level of education and APOE status. Significant clusters (p<0.005, TFCE) from the "*higher-risk" < "lower-risk*" contrast are projected onto four sagittal slices. TFCE values are indicated on a red-yellow colour bar.

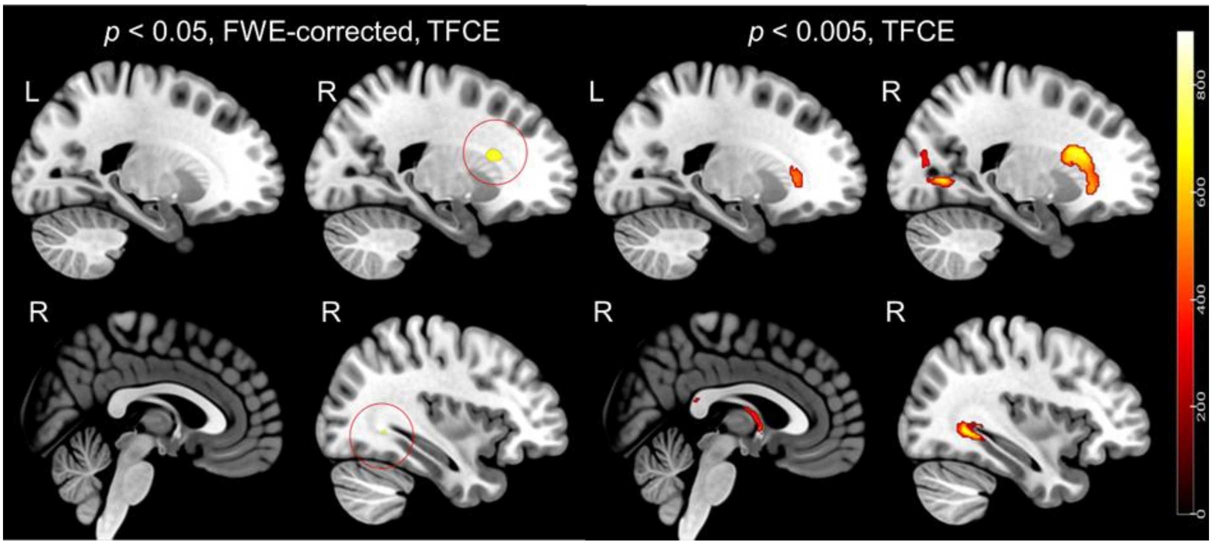

Supplementary Figure 11 Results for white matter volume with APOE status as additional covariate. White matter volume was compared between groups in a *two-sample t-test* (SPM12) correcting for age, sex, level of education and APOE status. Significant clusters from the "*higher-risk"* < *"lower-risk*" contrast are projected onto four sagittal slices for both FWE-corrected (p<0.05, TFCE) and uncorrected results (p<0.005, TFCE). TFCE values are indicated on a red-yellow colour bar.

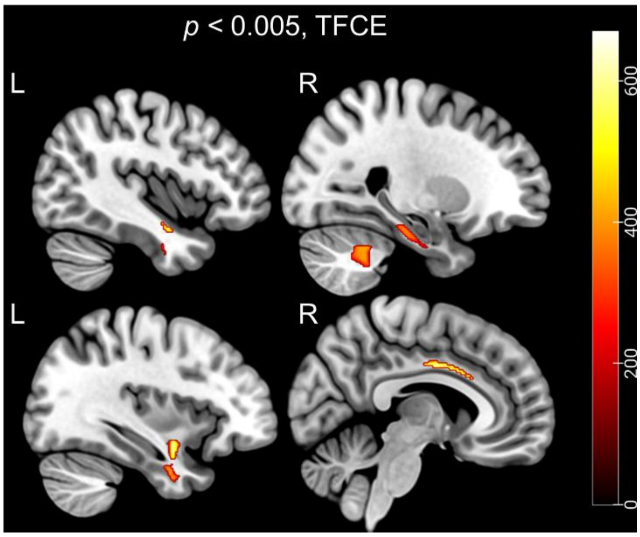

Supplementary Figure 12 Results for mean diffusivity values with APOE status as additional covariate. Mean diffusivity values were compared between groups in a two-sample t-test (SPM12) correcting for age, sex, level of education and APOE status. Significant clusters (p<0.005, TFCE) from the "lower-risk" < "higher-risk" contrast are projected into four sagittal slices. TFCE values are indicated on a red-yellow colour bar.

2.6 Replication with smoker status as additional covariates

Groups comparisons were replicated by adding two variables reflecting past and present smoker status (lifetime smoker and present smoker).

2.6.1 Cognition

	**ALL** ***N*=131**	**« Lower-risk » group** ***N*=92**	**« Higher-risk » group** ***N*=39**	**Comparisons**
**Episodic memory**	0.02 (1.00) [-2.81;1.93]	0.16 (0.98) [-2.49;1.93]	-0.26 (0.98) [-2.81;1.32]	*F*=2.607; *p*=0.109; *d*=0.198
**Executive functioning**	0.02 (0.99) [-3.04;2.46]	0.08 (1.00) [-2.24;2.46]	-0.11 (0.97) [-3.04;2.24]	*F*=0.034; *p*=0.854; *d*=0.023
**Attention-processing speed**	0.02 (0.99) [-1.87;2.09]	0.07 (1.01) [-1.87;2.09]	-0.09 (0.99) [-1.70;2.03]	*F*=0.015; *p*=0.902; *d*=0.015

Supplementary Table 6 Results of ANCOVAs comparisons for cognitive scores with smoker status as additional covariate. Data are mean (sd) [range] of standardised composite scores, reported for the whole sample and for each group. Groups were compared with ANCOVAs controlling for age, sex, level of education and smoker status. Effect sizes are specified with Cohen’s d.

2.6.2 Structural integrity

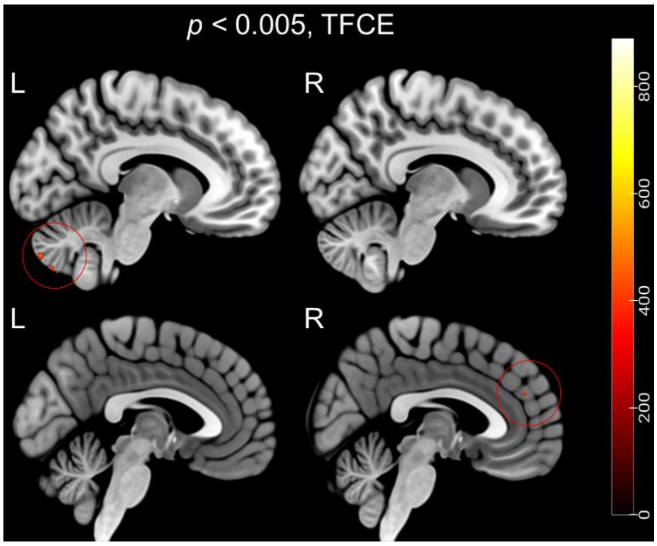

Supplementary Figure 13 Results for grey matter volume with smoker status as an additional covariate. Grey matter volume was compared between groups in a *two-sample t-test* (SPM12) correcting for age, sex, level of education and smoker status. Significant clusters (p<0.005, TFCE) from the "*higher-risk*" < "*lower-risk*" contrast are projected onto four sagittal slices. TFCE values are indicated on a red-yellow colour bar.

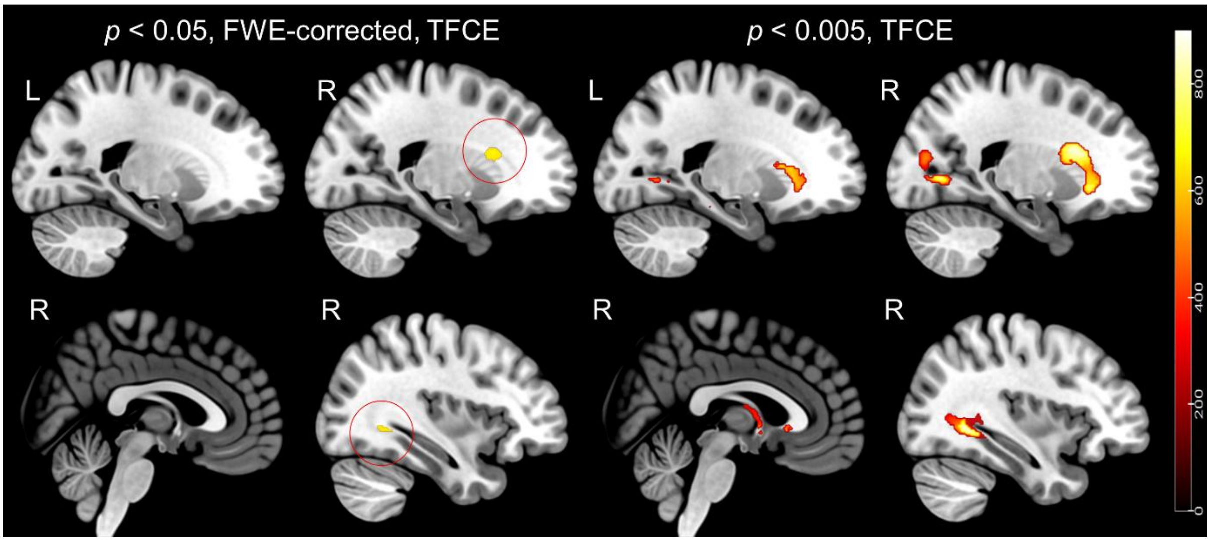

Supplementary Figure 14 Results for white matter volume with smoker status as an additional covariate. White matter volume was compared between groups in a *two-sample t-test* (SPM12) correcting for age, sex, level of education and smoker status. Significant clusters from the "*higher-risk"* < *"lower-risk*" contrast are projected onto four sagittal slices for both FWE-corrected (p<0.05, TFCE) and uncorrected results (p<0.005, TFCE). TFCE values are indicated on a red-yellow colour bar.

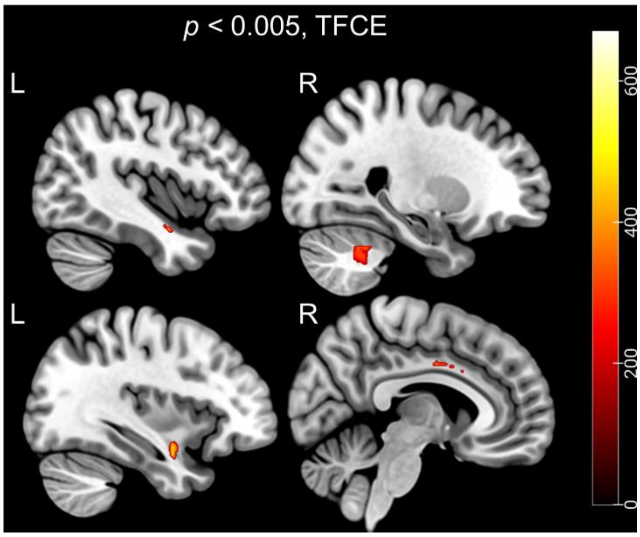

Supplementary Figure 15 Results for mean diffusivity values with smoker status as an additional covariate. Mean diffusivity values were compared between groups in a *two-sample t-test* (SPM12), correcting for age, sex, level of education and smoker status. Significant clusters (p<0.005, TFCE) from the "*lower-risk"* < *"higher-risk*" contrast are projected into four sagittal slices. TFCE values are indicated on a red-yellow colour bar.

2.6.3 Amyloid burden

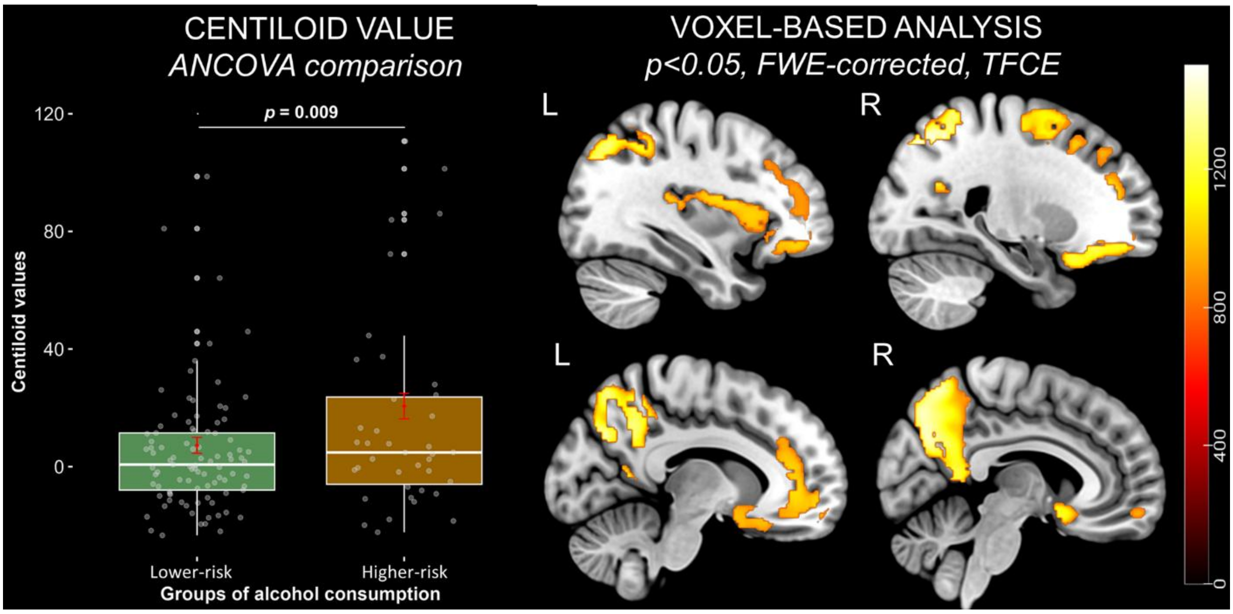

Supplementary Figure 16 Results for late AV45 SUVr with smoker status as additional covariate. Left: Boxplots illustrate the distribution of Centiloid values within each group of alcohol consumption. Individual data points are shown as jittered dots to display the distribution. Estimated marginal means and standard errors are in red. Groups were compared on a Type III ANCOVA correcting for age, sex, level of education, smoker status and APOE status. **Right:** Mean AV45 SUVr values were compared between groups in a *two-sample t-test* (SPM12) correcting for age, sex, level of education, smoker status and APOE status. Significant clusters from the "*lower-risk*" < *"higher-risk*" contrast are projected onto four sagittal slices for FWE-corrected results (*p*<0.05, TFCE). TFCE values are indicated on a red-yellow colour bar.

2.7 References

Klunk, W. E., Koeppe, R. A., Price, J. C., Benzinger, T. L., Devous, M. D., Jagust, W. J., Johnson, K. A., Mathis, C. A., Minhas, D., Pontecorvo, M. J., Rowe, C. C., Skovronsky, D. M., & Mintun, M. A. (2015). The Centiloid Project : Standardizing quantitative amyloid plaque estimation by PET. *Alzheimer's & dementia : the journal of the Alzheimer's Association*, *11*(1), 1—15.e1—4. https://doi.org/10.1016/j.jalz.2014.07.003

Navitsky, M., Joshi, A. D., Kennedy, I., Klunk, W. E., Rowe, C. C., Wong, D. F., Pontecorvo, M. J., Mintun, M. A., & Devous Sr., M. D. (2018). Standardization of amyloid quantitation with florbetapir standardised uptake value ratios to the Centiloid scale. *Alzheimer's & Dementia*, *14*(12), 1565-1571. https://doi.org/10.1016/j.jalz.2018.06.1353
