# Supplementary material for: Higher amyloid deposition and lower white matter volume in cognitively healthy seniors exceeding French alcohol recommendations
Source: eBioMedicine. 2026 Jul 15;130:106376. doi: 10.1016/j.ebiom.2026.106376 (PMC13382593; doi:10.1016/j.ebiom.2026.106376)
Supplement: Collab group [file mmc2.docx]

**The Medit-Ageing Research Group**

| **First Names** | **Surnames** |
| --- | --- |
| Eider | Arenaza-Urquijo |
| Emiliano | Albanese |
| Florence | Allais |
| Claire | André |
| Sebastian | Baez-Lugo |
| Mohamed | Bahi |
| Nikita | Belly |
| Maelle | Botton |
| Gaël | Chételat |
| Anne | Chocat |
| Fabienne | Collette |
| Robin | de Flores |
| Vincent | de La Sayete |
| Marion | Delarue |
| Stéphanie | Egret |
| Rawda | El Sadawy |
| Eglantine | Ferrand Devoue |
| Eric | Frison |
| Idir | Hamdidouche |
| Marc | Heidmann |
| Thibaut | Jorand |
| Agathe | Joret |
| Perla | Kaliman |
| Olga | Klimecki |
| Elizabeth | Kuhn |
| Brigitte | Landeau |
| Julie | Lebahar |
| Gwendoline | Ledu |
| Valérie | Lefranc |
| Antoine | Lutz |
| Marine | Manard |
| Natalie L. | Marchant |
| Sara | Martinez |
| Florence | Mezenge |
| Laurence | Michel |
| Inès | Moulinet |
| Valentin | Ourry |
| Christophe | Philips |
| Géraldine | Poisnel |
| Anne | Quillard |
| Géraldine | Rauchs |
| Stéphane | Rehel |
| Charlotte | Reid |
| Laura | Richert |
| Eric | Salmon |
| Corinne | Schimmer |
| Siya | Sherif |
| Delphine | Smagghe |
| Rhonda | Smith |
| Clémence | Tomadesso |
| Edelweiss | Touron |
| Patrik | Vuilleumier |
| Cédric | Wallet |
| Caitlin | Ware |
| Miranka | Wirth |
